# MicroRNA-129-5p-regulated microglial expression of the surface receptor CD200R1 controls neuroinflammation

**DOI:** 10.1016/j.jbc.2021.101521

**Published:** 2021-12-22

**Authors:** Vikas Singh, Shaivya Kushwaha, Jamal Ahmad Ansari, Siddhartha Gangopadhyay, Shubhendra K. Mishra, Rajib K. Dey, Ashok K. Giri, Satyakam Patnaik, Debabrata Ghosh

**Affiliations:** 1 Immunotoxicology Laboratory, Food, Drug & Chemical Toxicology Group and Nanomaterial Toxicology Group, CSIR-Indian Institute of Toxicology Research (CSIR-IITR), Lucknow, Uttar Pradesh, India; 2 Academy of Scientific and Innovative Research (AcSIR), Ghaziabad, India; 3 Developmental Toxicology Laboratory, Systems Toxicology & Health Risk Assessment Group, CSIR-Indian Institute of Toxicology Research (CSIR-IITR), Lucknow, Uttar Pradesh, India; 4 Molecular Genetics Division, CSIR-Indian Institute of Chemical Biology (CSIR-IICB), Kolkata, West Bengal, India; 5 Water Analysis Laboratory, Nanomaterial Toxicology Group, CSIR-Indian Institute of Toxicology Research, Lucknow, Uttar Pradesh, India

**Keywords:** microglia, miR-129-5p, CD200R1, arsenic, miRNA, neuroinflammation, cytokine, neuroimmunology, CEBPβ, CCAAT/enhancer-binding protein β, CSIR-IITR, CSIR–Indian Institute of Toxicology Research, DNMT1, DNA methyl transferase1, EAE, experimental autoimmune encephalomyelitis, EAU, experimental autoimmune uveoretinitis, FBS, fetal bovine serum, gDNA, genomic DNA, HDAC-1, histone deacetylase-1, IgSF, immunoglobulin superfamily, IP, immuneprecipitated, miRNA, microRNA, PBMC, peripheral blood mononuclear cell, SCI, spinal cord injury, SDS, sodium dodecyl sulfate, TLDA, TaqMan low-density array, WT, wild type

## Abstract

CD200R1 is an inhibitory surface receptor expressed in microglia and blood macrophages. Microglial CD200R1 is known to control neuroinflammation by keeping the microglia in resting state, and therefore, tight regulation of its expression is important. CCAAT/enhancer-binding protein β (CEBPβ) is the known regulator of CD200R1 transcription. In the present study, our specific intention was to find a possible posttranscriptional regulatory mechanism of CD200R1 expression. Here we investigated a novel regulatory mechanism of CD200R1 expression following exposure to an environmental stressor, arsenic, combining *in silico* analysis, *in vitro*, and *in vivo* experiments, as well as validation in human samples. The *in silico* analysis and *in vitro* studies with primary neonatal microglia and BV2 microglia revealed that arsenic demethylates the promoter of a microRNA, miR-129-5p, thereby increasing its expression, which subsequently represses CD200R1 by binding to its 3′-untranslated region and shuttling the *CD200R1* mRNA to the cytoplasmic-processing body in mouse microglia. The role of miR-129-5p was further validated in BALB/c mouse by stereotaxically injecting anti-miR-129. We found that anti-miR-129 reversed the expression of CD200R1, as well as levels of inflammatory molecules IL-6 and TNF-α. Experiments with a CD200R1 siRNA-induced loss-of-function mouse model confirmed an miR-129-5p→CD200R1→IL-6/TNF-α signaling axis. These main findings were replicated in a human cell line and validated in human samples. Taken together, our study revealed miR-129-5p as a novel posttranscriptional regulator of CD200R1 expression with potential implications in neuroinflammation and related complications.

CD200R1 belongs to the immunoglobulin superfamily (IgSF) (1), and it uses CD200 as the natural ligand (2). The human and mouse CD200R1 shows a similar distribution pattern with the highest expression in myeloid-derived cells such as macrophage, microglia, neutrophils as well as subsets of T and B cells (1, 3, 4).

CD200R1 is primarily involved in the inflammatory response. Cross-linking of CD200R1 with CD200-Fc fusion protein reduces the inflammation in arthritis model animals (5), and inhibition of CD200R1 by antibody reduces virus-induced cytokine storm (6). Interaction of CD200R1 with CD200 helps in tumor progression, and interventions are ongoing to disrupt this interaction (7, 8). High expression of CD200R1 has been observed in mammary repopulating units with stem-like properties (9). It also modulates bone mass by altering the osteoclast differentiation and plays a vital role in the regulation of mast cell functions (3, 10).

Microglial CD200R1 is closely associated with neuroinflammatory diseases (4, 11). MPTP and 6-OHDA-induced models of Parkinson’s disease show decreased CD200R1 expression, higher microglial activation, and increased TNF-α and IL-1β secretion with progressive degeneration of dopaminergic neurons (12, 13). The presence of CD200R1 agonists reduces LPS-induced neuroinflammation, while its blockade by anti CD200R1 antibody abrogates the effect (14). CD200R1 mRNA expression in Alzheimer’s disease brain sample is low (15), and IL4 treatment-mediated increase in low basal CD200R1 expression in human microglia is a potential therapeutic strategy (15). Upregulated CD200R1 ligand, CD200, and its agonist, CD200Fc attenuate demyelination in murine Experimental Autoimmune Encephalomyelitis (EAE) (16, 17), and Experimental Autoimmune Uveoretinitis (EAU) (18). Antibody-mediated blockade of CD200R1 in the Spinal Cord Injury (SCI) model deteriorates locomotor activity and aggravates demyelination and neuronal loss. Conversely, the administration of recombinant CD200-His reverses the effect (19). CD200R1 is also associated with microglia priming (20), antigen presentation (18), and cytokine production (21). Activation of CD200R1 slows down microglial migration and phagocytosis (22), whereas microglia isolated from CD200-deficient mouse model shows augmented phagocytosis and lysosomal activity (23). CD200R1 also promotes microglial proliferation (24, 25) and microglia-mediated neuronal death (26, 27), and the proliferation is reversed following recombinant CD200-His administration (19).

It is evident that CD200R1 is an important multifunctional protein, but the regulation of its expression is not well studied. Available literature describes the transcriptional regulation of CD200R1 expression, but posttranscriptional regulation is entirely unknown. The transcription factor CAAT Enhancer-Binding Protein Beta (CEBPβ) downregulates CD200R1 expression through histone deacetylase-1 (HDAC-1) (28). In contrast, PPAR-γ prevents the downregulation of CD200R1 in microglia upon inflammatory stimuli (29). The posttranscriptional regulation of gene expression is very important, and the regulatory molecules can be modulated by various environmental chemicals (30). MicroRNAs (miRNAs) are the most prominent posttranscriptional regulatory molecule, as evidenced by the fact that miRNAs regulate the expression of more than 60% of protein-coding genes in humans (31) by translational repression (32). Among various environmental chemicals, arsenic alters the miRNA profile in different experimental systems (33, 34).

The present study comprehensively sought to identify the mechanism of posttranscriptional regulation of CD200R1 expression, which can open new targets with therapeutic potential. We checked the expression of CD200R1 in the mouse brain as well as primary microglia following exposure to arsenic. With the help of TaqMan Low-Density Array (TLDA) for 641 miRNAs and using various *in silico* tools, specific miRNA was predicted that can potentially modulate CD200R1 expression. The binding of miRNA to CD200R1 was confirmed by using mutant constructs of CD200R1 3′-UTR. Our study was extended to an *in vivo* setting with knockdown of the miRNA and CD200R1 by injecting its specific anti-miRNA and *in vivo* ready siRNA respectively in the brain using the stereotaxic technique. Finally, the expressions of CD200R1, DNMT1, miRNA, and cytokine levels were correlated in peripheral blood mononuclear cells (PBMCs) of arsenic-exposed human subjects. Overall, the findings revealed that miR-129-5p is a novel posttranscriptional regulator of CD200R1 that controls neuroinflammation.

## Results

### CD200R1 expression is downregulated by arsenic as well as LPS

**Figure 1:**
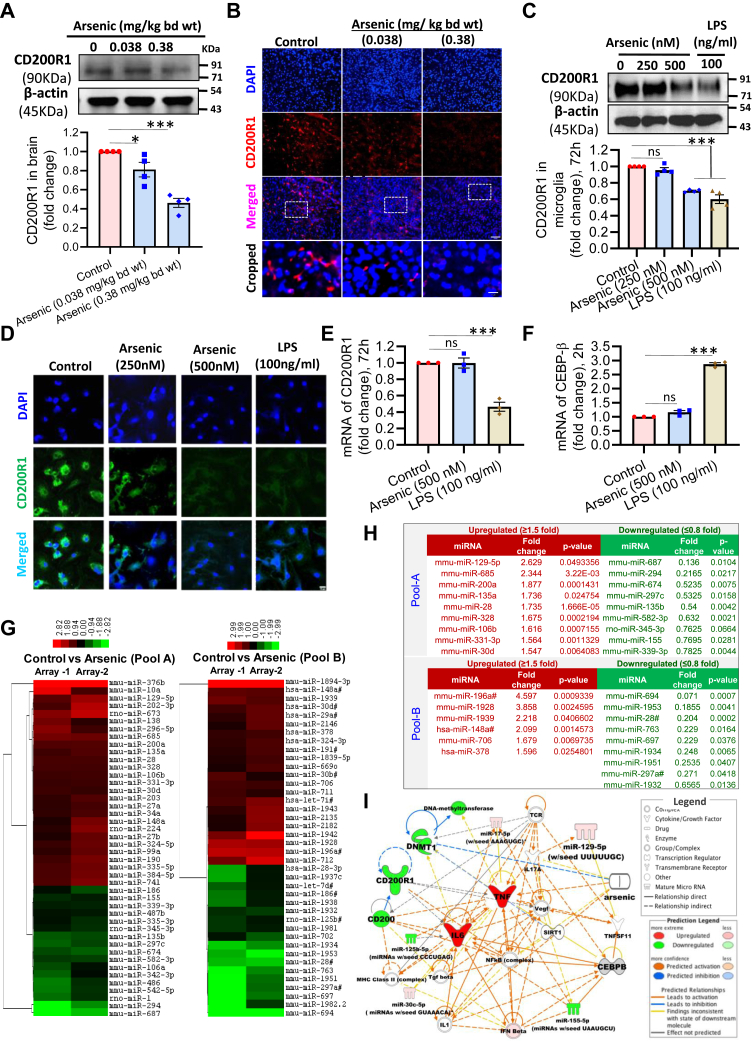
**Effect of arsenic stress on the expression of microglial CD200R1.** BALB/c mice were exposed to sodium arsenite (arsenic) for 2 months and checked the expression of CD200R1 in the whole brain (n = 4 mice/group). *A*, Western blots, (*B*) immunostaining of CD200R1 in brain section. Scale bar: 50 μm for uncropped images and 11 μm for the cropped images. Mouse primary neonatal microglia were treated with arsenic as well as LPS *in vitro* for 72 h, and CD200R1 expression was checked (n = 4). *C,* Western blot, (*D*) immunostaining of CD200R1 in mouse primary neonatal microglia. Scale bar: 10 μm. The levels of mRNA of (*E*) CD200R1 were measured 72 h following treatment with arsenic and LPS in primary microglia and, (*F*) CEBPβ was measured (n = 3) in primary microglia 2 h following treatment with arsenic and LPS. Another set of cells was treated *in vitro* with arsenic, and Taqman Low-Density Array (TLDA) was run to detect the changes in the global miRNA profile (n = 2). *G*, fold change in the miRNA level was presented as a heatmap, (*H*) miRNA upregulated more than 1.5-fold and downregulated more than 0.8-fold was presented in the table; (*I*) Network generated by IPA with the up- and downregulated genes and miRNA in arsenic-treated microglia indicated a possible relationship between CD200R1 and miR-129-5p. The *symbols* used in the network map are shown in the legend. “n” denotes the number of independent study for *in vitro* experiments. Bar graphs represent mean ± SEM. “*p*” denotes the level of significance in comparison to control; ∗*p* < 0.05, ∗∗*p* < 0.01, ∗∗∗*p* < 0.001; ns, nonsignificant.

The expression of microglial CD200R1 is associated with its activation status (28). Earlier, we have shown that *in vivo* arsenic exposure activates microglia (35, 36). To test whether arsenic modulates the expression of CD200R1, we analyzed its expression in the brain lysate of arsenic-exposed mice with Western blot. A significant decrease in the CD200R1 expression (∼0.8-fold) in 0.038 and (∼0.4-fold) 0.38 mg/kg body weight arsenic-treated group compared with the control was observed (Fig. 1*A*). We also checked whether CD200R1 is exclusively expressed in microglia by coimmunolabeling CD200R1 with other cell-type-specific markers. CD200R1 did not colocalize with GFAP (astrocyte marker), MAP-2 (Neuronal marker), and MBP (oligodendrocyte marker) but for the microglial marker, Iba1. Colocalization of CD200R1 and Iba1 confirms the exclusive expression of CD200R1 in microglia or perivascular macrophages in the brain (Fig. S1*A*). The Western blot data corroborated with immunostaining of CD200R1 in brain sections (Figs. 1*B* and S1*B*). We also checked the CD200R1 expression in primary neonatal microglia exposed *in vitro* to nontoxic doses (250 and 500 nM) of arsenic for 72 h (Fig. S1*C*). Microglia exposed to LPS was used as a positive control (28). Arsenic (500 nM) and LPS (100 ng/ml) both significantly reduced the expression of CD200R1 (∼0.7- and 0.6-fold, respectively) (Fig. 1*C*). A similar pattern of CD200R1 expression was observed in immunofluorescence staining of CD200R1 following *in vitro* treatment (Fig. 1*D*). Henceforth, in all *in vitro* experiments, 500 nM arsenic and 100 ng/ml LPS were used.

### Suppression of CD200R1 is differentially regulated by arsenic and LPS

Our first aim was to elucidate whether arsenic affects the expression of CD200R1 at the transcriptional or the posttranscriptional level. The changes in levels of CD200R1 and CEBPβ, the known transcriptional regulator of CD200R1, were studied in primary neonatal microglia exposed to arsenic and LPS *in vitro* by real-time PCR (qRT-PCR) and Western blotting. Though the protein levels of CD200R1 decreased following 72 h *in vitro* arsenic exposure (Fig. 1, *C* and *D*), its mRNA level showed no significant change compared with control. But in LPS-exposed microglia, the mRNA level of CD200R1 decreased significantly following 72 h treatment (Fig. 1*E*). The mRNA level of CD200R1 transcriptional inhibitor CEBPβ did not show any significant change following 2 h *in vitro* arsenic treatment. But it increased significantly in the LPS group (∼2.8-fold) compared with control, following 2 h exposure (Fig. 1*F*). These nonparallel changes observed in the levels of CD200R1 protein and mRNA, and the differential expression of CEBPβ in arsenic and LPS-exposed group, points toward the possibility that CD200R1 expression is posttranscriptionally regulated. MiRNAs are the potential posttranscriptional regulators of gene expression. Therefore, we screened for miRNAs in primary microglia following *in vitro* arsenic (500 nM) treatment for 72 h. The list of miRNAs showing significant differential expression is presented in the heatmap (Fig. 1*G*). A total of 15 significantly upregulated (≥1.5-fold) and 18 significantly downregulated (≤0.8-fold) miRNAs were identified (Fig. 1*H*). We uploaded the list of miRNA and mRNA of our interest into Ingenuity pathway analysis and built a network to identify any possible relationships between various molecules. We could not find any direct link between CD200R1 and miR-129-5p (Fig. 1*I*).

### MicroRNA-129-5p is involved in the posttranscriptional regulation of CD200R1 expression in mouse and human microglia

**Figure 2:**
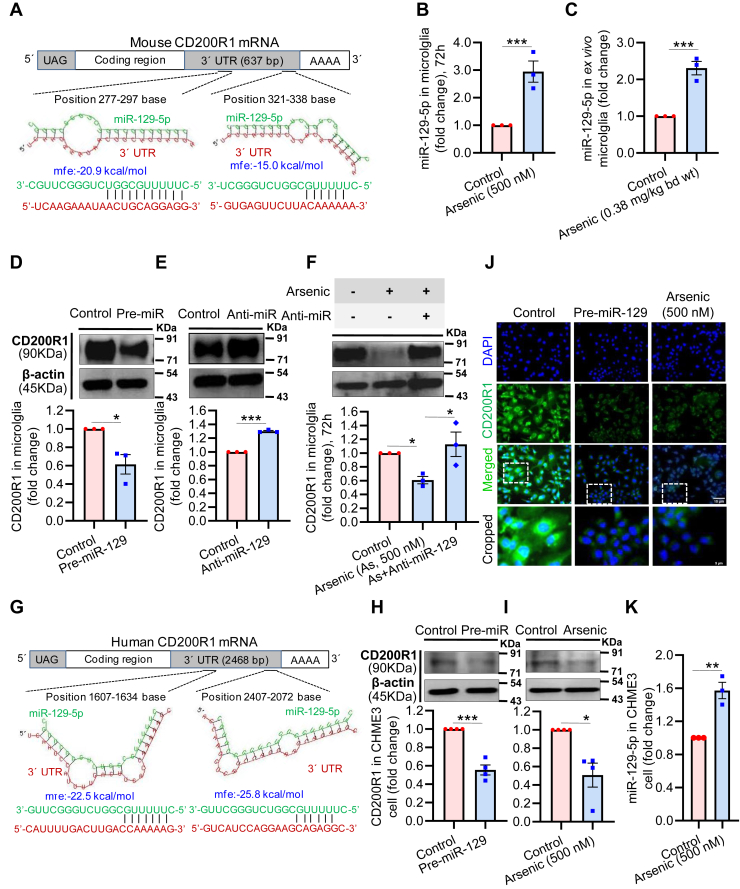
**Involvement of miR-129-5p in the regulation of CD200R1 expression.***A*, *in silico* analysis using TargetScan and RNAhybrid predicted the two putative miR-129-5p binding sites (277–297 bases and 321–338 bases) in the 3′UTR of mouse CD200R1 mRNA. *B*, *in vitro* arsenic exposure increased the level of miR-129-5p in neonatal microglia (n = 3); (*C*) similar increase was observed in *ex vivo* microglia following *in vivo* arsenic exposure (n = 3 mice/group). *D*, *in vitro* transfection of pre-miR-129 for 72 h reduced the expression of CD200R1 (n = 3); (*E*) whereas anti-miR-129 transfection increased the expression of CD200R1 (n = 3); (*F*) Microglia were treated with arsenic followed by anti-miR-129 transfection showed reversal effect of arsenic on CD200R1 expression (n = 3). *G*, *in silico* analysis also predicted two putative miR-129-5p binding sites (1607–1634 bases and 2047–2072 bases) in the 3′UTR of human CD200R1 mRNA. Reduction in CD200R1 expression was observed in CHME3 cells following (*H*) *in vitro* transfection of pre-miR-129 (n = 4) and (*I*) *in vitro* arsenic exposure for 72 h (n = 4). *J*, immunostaining of human CD200R1 showed reduced immunoreactivity following pre-miR-129 and arsenic treatment. Scale bar: 15 μm for uncropped images and 5 μm for the cropped images. *K*, *in vitro* arsenic exposure increased the level of miR-129-5p in CHME3 cells. “n” denotes the number of independent study for *in vitro* experiments. Bar graphs represent mean ± SEM. “*p*” denotes the level of significance in comparison to control; ∗*p* < 0.05, ∗∗*p* < 0.01, ∗∗∗*p* < 0.001; ns, nonsignificant.

*In silico* analysis with TargetScan, RNA22, and RNA hybrid showed that miR-129-5p targets CD200R1 by binding to its 3′-UTR. Two putative binding sites of miR-129-5p (bp 277–297 and 321–338) were identified in the 3′-UTR of mouse CD200R1 mRNA (Fig. 2*A*). Individual analysis showed that the levels of miR-129-5p were significantly elevated in the primary microglia exposed to arsenic *in vitro* (∼3-fold) (Fig. 2*B*) and also in *ex vivo* (∼2.3-fold) microglia isolated from control and 60 days arsenic-exposed animals (Fig. 2*C*). Thus, exposure to arsenic alters the level of miR-129-5p. Next, we studied the role of miR-129-5p in the regulation of CD200R1 expression. First, we determined the dose and efficiency of pre- and anti-miR-129 and observed that pre-miR (10 nM) and anti-miR (100 nM) transfection increased and decreased the miR-129-5p expression, respectively (data not shown). The CD200R1 protein levels decreased significantly (∼0.6-fold) in neonatal primary microglia exposed to pre-miR-129 for 72 h *in vitro* (Fig. 2*D*), and the exposure to anti-miR-129 increased the expression (∼1.3-fold) (Fig. 2*E*). A similar effect of anti-miR-129 was observed in arsenic exposed microglia where anti-miR-129 reversed the arsenic-induced downregulation of CD200R1 (∼0.6-fold in arsenic and ∼1.1-fold in arsenic + anti-miR group) (Fig. 2*F*). This supports our notion that miR-129-5p regulates CD200R1 expression posttranscriptionally. To check whether miR-129-5p function is similar across the species, we performed *in silico* analysis and found that miR-129-5p has two binding sites (bp 1607–1634 and 2047–2972) in the 3′-UTR of human CD200R1 (Fig. 2*G*) similar to that found in mouse. Human microglial cell line CHME3 exposed to arsenic or pre-miR-129 for 72 h showed that the protein level of CD200R1 was significantly downregulated (∼0.56-fold for pre-miR and ∼0.51-fold for arsenic) (Fig. 2, *H* and *I*). Lower immunoreactivity of CD200R1 in CHME3 cells with pre-miR-129 and arsenic showed an expression pattern (Fig. 2*J*) similar to that observed in Western blot analysis. Increased expression of the miR-129-5p (∼0.1.6-fold) following *in vitro* arsenic exposure in CHME3 cells (Fig. 2*K*) also supports the involvement of arsenic-induced upregulation of miR-129-5p across the species.

### MicroRNA-129-5p induces translational repression by guiding CD200R1 to the cytoplasmic processing body (P-body)

**Figure 3:**
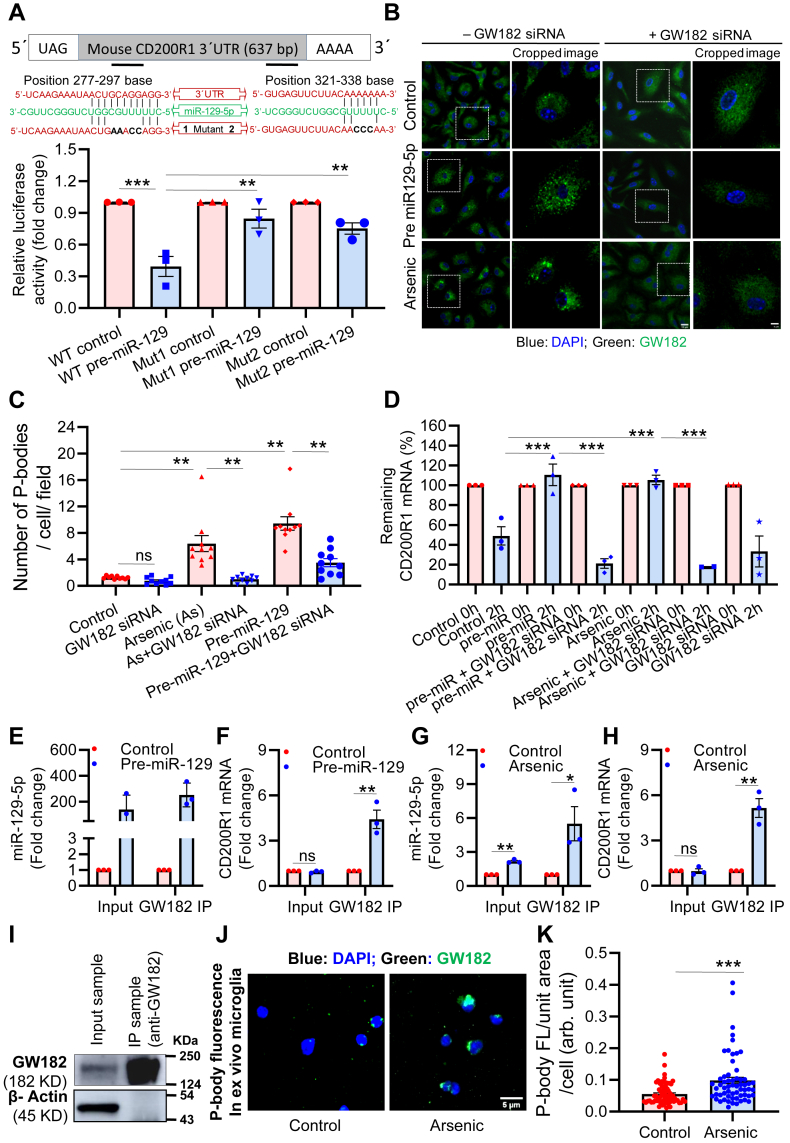
**MiR-129-5p guides CD200R1 mRNA to p-bodies.***A*, luciferase reporter assay using mouse CD200R1 3′-UTR constructs (pMIR-WT, pMIR-Mut1 & pMIR-Mut2) cloned in pMIR-REPORT vector confirmed that both the predicted binding sites are involved in miR-129-5p-mediated repression of CD200R1 (n = 3). *B* and *C*, P-bodies were stained with GW182 antibody in primary neonatal microglia following pre-miR-129 and arsenic exposure for 72 h. It showed increased puncta formation in the cytoplasm. Scale bar: 10 μm for uncropped images and 4 μm for the cropped images. *D*, microglia were treated with pre-miR-129 and arsenic for 72 h followed by actinomycin D (5 μg/ml) treatment for 0, 2 h. Cells were harvested, RNA isolated, and run for real-time PCR to detect CD200R1. Pre-miR-129 and arsenic protected CD200R1 mRNA from degradation. To detect the presence of miR-129-5p and CD200R1 mRNA in p-bodies, GW182 and pre-miR-129 were overexpressed in BV2 cells (n = 3) and the levels of (*E*) miR-129-5p and (*F*) CD200R1 mRNA were measured in GW182 immunoprecipitated (GW182 IP) samples. Similarly, GW182 overexpressed BV2 cells were treated with arsenic (n = 3) and level of (*G*) miR-129-5p, and (*H*) CD200R1 mRNA were measured in GW182 IP samples. Levels of both miR-129-5p and CD200R1 mRNA in the GW182 IP samples were found to be significantly high (n = 3). *I*, to check the contamination-free IP of GW182, the GW182 protein was immunoprecipitated using an anti-GW182 antibody and processed for WB analysis. Sixty microgram lysate (7.5% of input for IP [800 μg]) loaded in lane-1 and 12 μl (60% of the total IP elution volume [20 μl]) loaded in lane-2. The absence of the β-actin band in IP lane shows the contamination free IP of GW182 (n = 2). Formation of P-body *in vivo* was also checked in *ex vivo* microglia isolated from mouse using GW182 immunostaining. CD200R1-associated fluorescence was quantitated in “Image J”. *J*, representative immunofluorescence stained microglia, (*K*) scatter plots representing quantitative analysis of GW182 associated immunofluorescence. (n = 4 mice/group; total cells in control group-67 and in arsenic group-60). Scale bar: 5 μm. “n” denotes the number of independent study for *in vitro* experiments. Bar graphs represent mean ± SEM. “*p*” denotes the level of significance in comparison to control; ∗*p* < 0.05, ∗∗*p* < 0.01, ∗∗∗*p* < 0.001; ns, nonsignificant.

To confirm the binding of miR-129-5p at the two predicted sites of the 3′-UTR of mouse CD200R1 mRNA, we generated mutant constructs for the two putative binding sites. Mutant 1 (bp 277–297) and mutant 2 (bp 321–338) were constructed by mutating bases in the 3′-UTR of mouse CD200R1 mRNA (Mut1: GCAGG to AAACC; Mut2: AAA to CCC) that binds to the seed sequence of the miRNA as shown in Fig. S2, *A*–*C*. To confirm the binding and translational repression of CD200R1 mRNA by miR-129, we performed luciferase assays. HEK293 cells pretreated with or without pre-miR-129 for 8 h were transfected with wild-type (WT) and mutant (Mut) pMiR-constructs. Forty-eight hours posttransfection, the luciferase activity had significantly decreased in the WT pretreated with miRNA (∼0.35-fold). But in the mutants, the luciferase activity had reversed (Mut1: 0.83-fold and Mut2: 0.73-fold) compared with the WT pre-miRNA-129. (Fig. 3*A*). The increased luciferase activity in the mutants compared with WT with pre-miR confirms that miRNA-129-5p binds to the CD200R1 mRNA. It also shows that both sites are involved in the posttranscriptional regulation of CD200R1 expression. MicroRNA induces translational repression by binding to its target mRNA and guiding it into P-bodies, whereupon they are inaccessible to translational machinery. Therefore, we studied whether there was an increase in the formation of P-bodies with GW182 immunostaining, one of the essential components of P-bodies. In BV-2 cells, both pre-miR-129 and arsenic both induced the formation of P-bodies (shown in Fig. 3, *B* and *C*) visible as the punctate fluorescence of GW182. Conversely, GW182-specific siRNA inhibited the puncta formation of GW182 (Fig. 3, *B* and *C*). GW182 siRNA significantly inhibited GW182 protein levels in BV2 cells to 40% showing its significant inhibitory efficiency (Fig. S2*D*).

CD200R1 mRNA guided into the P-bodies becomes inaccessible to the degradation machinery. To test this, we studied the rate of CD200R1 mRNA decay in the presence or absence of GW182 siRNA following P-body induction either by pre-miR-129 or arsenic. After P-body induction, followed by actinomycin D (Act-D) treatment, microglial cells were harvested at 0 h and 2 h time points, and remaining CD200R1 mRNA level was measured with qRT-PCR. In the control group, approximately 50% (∼0.49-fold) mRNA decay was observed after 2 h, whereas, in pre-miR-129 and arsenic-treated groups, there was no significant change in the mRNA levels (∼1.1-fold). But, there was significant mRNA decay in the GW182 siRNA group (Fig. 3*D*). Prevention of CD200R1 mRNA from degradation in pre-miR-129 and arsenic-treated group is possibly due to the inclusion of miR-129-5p bound CD200R1 mRNA into the p-bodies. In the siRNA group, knockdown of GW182 inhibited p-body formation resulting in the degradation of the CD200R1 mRNA (Fig. 3*D*).

To check the localization of miR-129-5p and CD200R1 mRNA in the p-bodies, GW182 and pre-miR-129 were overexpressed in BV2 cells. P-bodies were immuneprecipitated (IP) with the GW182 antibody, and the total RNA was isolated. The levels of miR-129-5p and CD200R1 mRNA were evaluated by qRT-PCR. As the miR-129 was overexpressed, its level was high in the input and the IP samples (Fig. 3*E*). A significant enrichment (∼4.5-fold) of CD200R1 mRNA was observed in the IP samples (Fig. 3*F*). Similarly, p-bodies were also IP from arsenic (1 μM) treated BV2 cell lysate followed by qRT-PCR to detect the level of CD200R1 and miR-129-5p. We used 1 μM arsenic to treat BV2 cells as a significant increase in miR-129-5p level was observed in 1 μM arsenic but not in the 500 nM treatment group (Fig. S2*E*). Significant enrichment of miR-129-5p (∼5.5-fold) (Fig. 3*G*) and CD200R1 mRNA (∼5.2 fold) (Fig. 3*H*) was also observed in the IP samples of the arsenic-treated group. The enrichment of miR-129-5p and CD200R1 mRNA in the IP samples in both the pre-miR-129 and arsenic-treated groups confirms that miR-129-5p binds to CD200R1 mRNA and brings it to the p-body, thereby inducing translation repression. We have also checked the enrichment of protein in the IP sample by Western blot analysis using the GW182 antibody. Thicker band of GW182 and absence of β-actin in the IP sample showed efficient immunoprecipitation of the P-body (Fig. 3*I*). Interestingly, similar to *in vitro* formation of p-body, *ex vivo* microglia isolated from arsenic-exposed animals also showed a significantly higher level of GW182-associated fluorescence, showing an increase in p-body *in vivo* (Fig. 3, *J* and *K*). The Pearson correlation analysis revealed an inverse relation between the number of p-bodies and the level of CD200R1 (Fig. S2*F*).

### Arsenic decreases the methylation of the promoter of miR-129-5p by downregulating DNMT1

**Figure 4:**
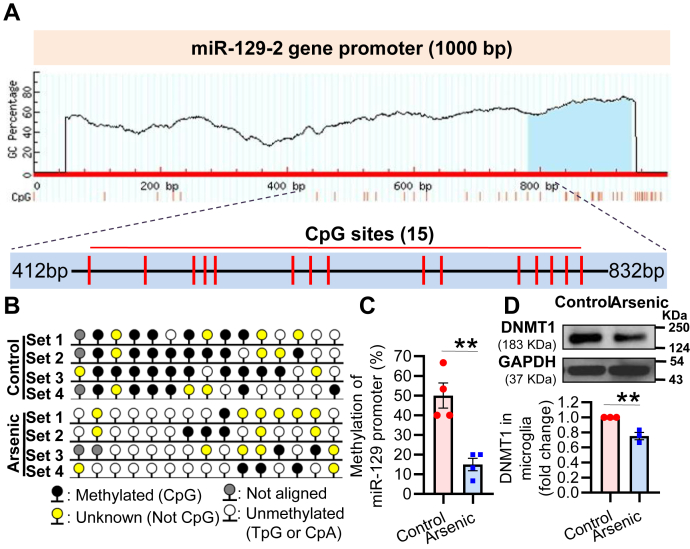
**Effect of arsenic on the methylation status of the miR-129-2 promoter and DNMT1 expression in primary neonatal microglia.***A*, *in silico* analysis, using MethPrimer software, predicted 15 CpG sites in the promoter region of miR-129-5p (bp 412−bp 832). *B*, bisulfite sequence of the promoter region of miR-129-5p was performed, and a methylation map of the CpG sites was generated using the MethPrimer software. *C*, the methylation status was quantitated and represented in a histogram (n = 4 samples/group). *D*, arsenic treatment for 72 h reduced the expression of DNMT1 in primary microglia, which is shown by representative Western blot and its densitometric analysis presented as a histogram (n = 3). “n” denotes the number of independent study for *in vitro* experiments. Bar graphs represent mean ± SEM. “*p*” denotes the level of significance in comparison to control; ∗*p* < 0.05, ∗∗*p* < 0.01, ∗∗∗*p* < 0.001; ns, nonsignificant.

Our study showed that miR-129-5p posttranscriptionally regulates the expression of CD200R1 following arsenic exposure. This raises the question: how does arsenic upregulate miR-129-5p? DNA methyl transferase1 (DNMT1) enzyme is responsible for the maintenance of methylation status (37) of the promoter of a gene and thereby regulates transcription. The miR-129 consists of two families of genes present on different chromosomes; miR-129-1 (Gene ID: 387237) located on chromosome 6qA3.3 and miR-129-2 (NCBI ID- 723953) located on chromosome 2pE1 (38). Both genes encode for miR-129-5p, but it is the miR-129-2 gene whose expression is regulated by DNA methylation. There is a high density of CpG sites upstream of the transcription start site (39). Arsenic is known to downregulate DNMT1 (40). Therefore, we searched for potential CpG sites 1000 bp upstream of the miR-129-2 gene transcriptional start site using Methprimer software (41). There were several CpG sites in the upstream sequence. Fifteen CpG sites (between 412 and 832 bp) were selected for bisulfite sequencing (Fig. 4*A*). Specific primers were designed to find out the methylation status of the promoter region following *in vitro* arsenic exposure. Our results showed ∼50% methylation of the promoter sequence in the control group, which decreased to ∼15% following 72 h arsenic treatment (Fig. 4, *B* and *C*). A significant decrease in DNMT1 expression (∼0.75-fold to control) was also observed following 72 h *in vitro* arsenic exposure (Fig. 4*D*) in primary neonatal microglia. Therefore, arsenic induces demethylation of the miR-129-5p promoter by suppressing the level of microglial DNMT1.

### *In vivo* inhibition of miR-129-5p reversed the arsenic-induced altered expression of CD200R1 and cytokine secretion, but simultaneous inhibition of miR-129-5p and CD200R1 did not have the same effect

**Figure 5:**
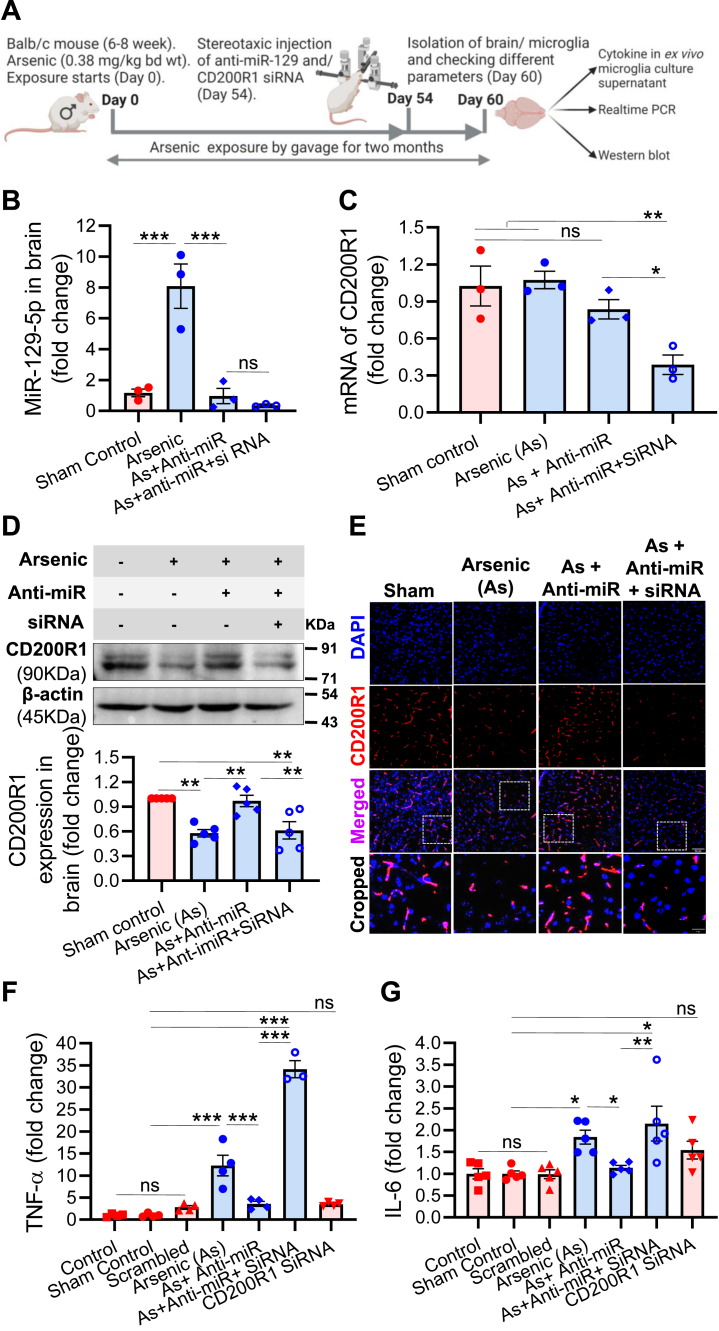
**Effect of *in vivo* CD200R1 and miR-129-5p inhibition on the level of microglial TNF-α and IL-6.** Animals were exposed to arsenic (0.38 mg/kg bd. wt.) for 2 months, and in the last week of exposure, animals were treated with CD200R1 siRNA and anti-miR-129 intracerebrally by stereotaxic method and sacrificed on 60^th^ day. *A*, experimental scheme, (*B*) exposure to arsenic increased the level of miR-129-5p, which was brought down by the treatment of anti-miR-129 (n = 3 mice/group). CD200R1 siRNA did not show any significant effect on the level of miR-129-5p. *C*, arsenic exposure did not alter the mRNA level of CD200R1 like *in vitro* compared with control, whereas siRNA significantly inhibited it. *D*, Western blot analysis showed that arsenic exposure inhibited CD200R1 protein, which was rescued by anti-miR (n = 5 mice/group). *E*, immunofluorescence staining of CD200R1 in brain sections (n = 3 mice/group). Scale bar: 50 μm for uncropped images and 17 μm for the cropped images. The effects of CD200R1 siRNA and anti-miR-129 on the level of (*F*) TNF-α (n = 3–4 mice/group) and (*G*) IL-6 (n = 5 mice/group) were measured in the *ex vivo* microglial culture supernatant. Bar graphs represent mean ± SEM. “*p*” denotes the level of significance in comparison to control; ∗*p* < 0.05, ∗∗*p* < 0.01, ∗∗∗*p* < 0.001; ns, nonsignificant.

We extended our study to validate the role of miR-129-5p *in vivo*. First, we ascertained the inhibitory efficiency of CD200R1 siRNA stereotaxically injected into the brain of unexposed mice on CD200R1 protein by Western blot using whole brain lysate (Fig. S3). We have also checked the inhibition of CD200R1 mRNA in different anatomical region of the brain by qRT-PCR. A region-specific inhibitory effect of CD200R1 siRNA was observed (Fig. S4). During the last week of 2 months of arsenic exposure, anti-miR-129 and CD200R1 siRNA were injected individually and in combination stereotaxically into mice brains (Fig. 5*A*). Scrambled (nontarget) siRNA was injected into the brains of control mice. miR-129-5p (∼8.1-fold) levels increased in the arsenic exposed group but, anti-miR-129 treatment significantly decreased the miR-129-5p level (∼0.97-fold) in the brain sample of arsenic-treated animal. CD200R1 siRNA did not alter the effect of anti-miR-129 (Fig. 5*B*). We measured the level of CD200R1 mRNA in the brain following arsenic, anti-miR, and siRNA treatment. CD200R1 siRNA decreased the CD200R1 mRNA levels significantly as expected compared with the controls. Whereas, most interestingly and supportive to our *in vitro* observation (Fig. 1*E*), the CD200R1 mRNA was not altered in the brain following *in vivo* treatment (Fig. 5*C*) even though arsenic could decrease the expression of CD200R1 protein significantly (∼0.58-fold) as detected in Western blot analysis (Fig. 5*D*). Anti-miR-129 treatment in arsenic-exposed animals significantly reversed the expression of CD200R1 (∼0.97-fold), whereas siRNA treatment neutralized the effect of anti-miR in arsenic-exposed animals (Fig. 5*D*). Brain sections were immunostained against CD200R1 (Fig. 5*E*), and its quantitative analysis (Fig. S5) supports the changes observed in the CD200R1 protein expression following arsenic, anti-miR, and/or siRNA treatment (Fig. 5*D*). The association of CD200R1 expression with neuroinflammation was ascertained by measuring the level of proinflammatory cytokines, TNF-α and IL-6 in the culture supernatant of *ex vivo* microglia. The elevated levels of TNF-α (∼12.28-fold) and IL-6 (∼1.84-fold) in the arsenic-exposed group decreased when treated with anti-miR-129-treated group (TNF-α ∼4.42- and IL-6 ∼1.07-fold) (Fig. 5, *F* and *G*). *In vivo* inhibition of CD200R1 by siRNA in arsenic and the anti-miR coexposed group increased the cytokine levels even beyond that of arsenic-exposed group (TNF-α ∼31.92-fold and IL-6 ∼2.15-fold). Cytokines in control, sham control, scrambled (nontarget control), and CD200R1 siRNA group did not show any significant alterations among themselves (Fig. 5, *F* and *G*). All these data reflect that the miR-129-5p posttranscriptionally regulates the CD200R1 expression and, in turn, controls the microglial inflammatory response in the mouse model.

### The altered expression of miR-129-5p, CD200R1, DNMT1, and cytokine expression observed in arsenic-exposed individuals corroborates with our experimental observations

**Figure 6:**
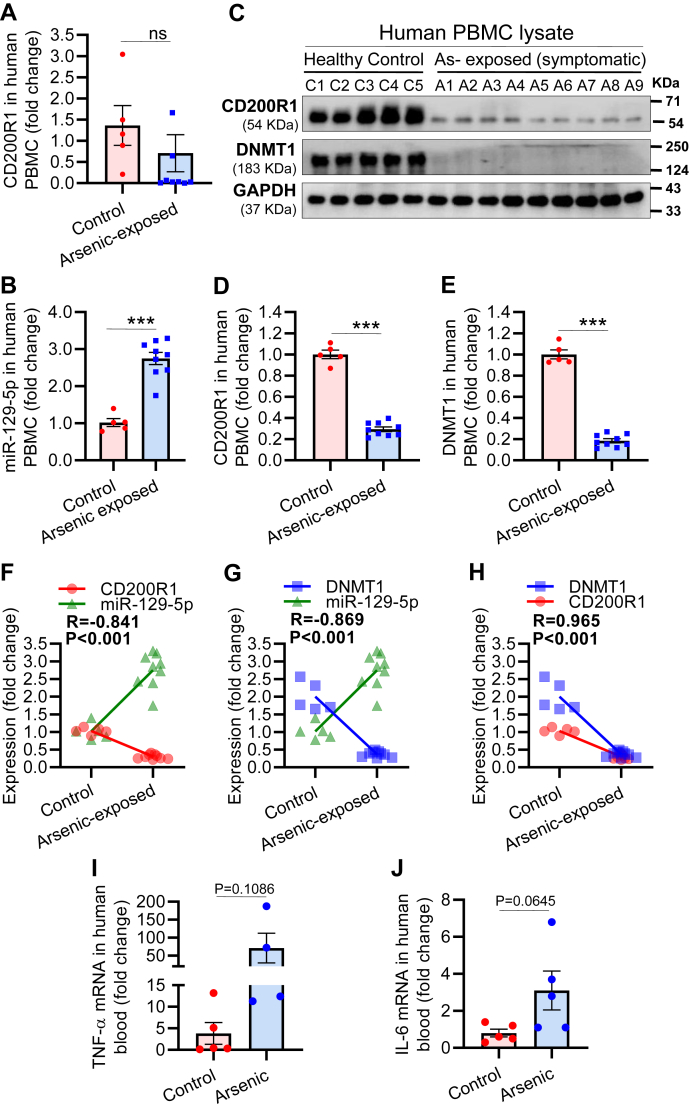
**Expression of CD200R1, miR-129-5p, DNMT1, and Cytokines in human blood.** PBMCs were isolated from the blood of unexposed (n = 5 individuals) and arsenic-exposed symptomatic individuals (n = 9 individuals). RNA was isolated, and the levels of (*A*) CD200R1 mRNA and (*B*) miR-129-5p were measured by real-time PCR. The expression levels of CD200R1 and DNMT1 were checked by Western blot analysis in PBMC lysate, (*C*) image of the Western blot and quantitative analysis of the expression of (*D*) CD200R1 and (*E*) DNMT1. Pearson correlation analysis revealed an inverse correlation between (*F*) miR-129-5p and CD200R1 as well as (*G*) miR-129-5p and DNMT1, whereas (*H*) CD200R1 and DNMT1 were directly correlated. “R” denotes Pearson correlation coefficient. For cytokine analysis, RNA was isolated from the whole blood of unexposed and arsenic-exposed individual followed by qRT-PCR for cytokines (n = 4–5 individuals/group). The level of mRNA was present as scatter plot for (*I*) TNF- α and (*J*) IL-6. An increased pattern in the level of IL-6/TNF-α mRNA was observed in arsenic-exposed individuals. Bar graphs represent mean ± SEM. “*p*” denotes the level of significance in comparison to control; ∗*p* < 0.05, ∗∗*p* < 0.01, ∗∗∗*p* < 0.001; ns, nonsignificant.

We further extended our study to validate our findings in clinical samples. For the study, we selected individuals (n = 9, average age ∼57 ± 11 years and weight ∼55 ± 12 kg) residing in the arsenic affected areas of Murshidabad district in the state of West Bengal, India, and showing clear raindrop pigmentation and symptoms of arsenicosis. Among selected individuals, four subjects also showed keratosis in addition. Murshidabad district is highly contaminated with arsenic. The range of arsenic concentrations in the groundwater in this region is 10 to 1000 μg/l (42). Control individuals (n = 5, average age ∼47 ± 10 years and weight ∼57 ± 8 kg) were selected from Lucknow in the state of Uttar Pradesh, India. The level of arsenic in groundwater in Lucknow is below the WHO permissible limit (http://jn.upsdc.gov.in/page/en/cpu). PBMCs were isolated from the blood of control and arsenicosis subjects. The Isolated PBMCs were divided into two groups, one set used for RNA extraction and another for protein extraction. We have checked the level of CD200R1 mRNA using real-time PCR primers, which can detect all four variants of CD200R1 mRNA and found a nonsignificant decrease (∼1.4-fold in control subjects and ∼0.71-fold in arsenicosis subjects) in their levels (Fig. 6*A*). In contrast, the CEBP-β level had increased nonsignificantly compared with control (Fig. S6). The level of miR-129-5p in the PBMCs of the arsenic-exposed population had increased significantly (∼2.5-fold) compared with the control group (Fig. 6*B*). Similar to our animal studies, we could detect a decrease in the expression of CD200R1 (∼0.3-fold) and DNMT1 (∼0.2-fold) in PBMCs isolated from arsenic-exposed individuals compared with the control group (Fig. 6, *C*–*E*). Pearson correlation analysis showed negative correlation between miR-129-5p and CD200R1 (R = −0.841) (Fig. 6*F*) and miR-129-5p and DNMT1 (R = −0.869) (Fig. 6*G*), but DNMT1 and CD200R1 showed positive correlation (R = +0.965) (Fig. 6*H*). The levels of TNF-α and IL-6 transcripts were higher compared with the controls as observed following qRT-PCR of RNA isolated from the blood (Fig. 6, *I* and *J*). Another interesting observation is that, among nine individuals, five individuals showed mild neuropathy with symptoms such as numbness, muscle cramp, and pain. In contrast, one individual showed high neuropathy with additional symptoms such as paresthesia, vibration joint sense, muscle wasting (Table S1).

## Discussion

The posttranscriptional regulation of CD200R1 is of great clinical interest as it is involved in neuroinflammation (4) and other cellular functions. In the present study, we strongly prove that miR-129-5p is involved in the posttranscriptional regulation of CD200R1 expression and neuroinflammation. To this end, we performed *in silico analysis* and *in vitro* experiments with primary microglia and both mouse and human cell lines. We further validated our results in animals (mice) and human samples.

Contact-dependent CD200R1-CD200 interaction between microglia and neuron plays a crucial role in the suppression of microglial neuroinflammation. The expression of CD200R1 decreases in various neuroinflammatory diseases such as AD, MS (15, 43) and in experimental disease models such as EAE, EAU, SCI, and PD (16, 18, 19, 44). Previously, we showed that expression of CD200 decreases in the arsenic-exposed mouse brain (35). In the present study, we showed that the expression of CD200R1, the cognate receptor of CD200, is reduced following arsenic exposure both *in vivo* and *in vitro*. This supports our previous studies on arsenic-induced microglial activation (35, 36). In recent years, the role of CD200R1 in neuroinflammation has been highly explored. Parallelly, miRNAs are emerging as the novel therapeutic targets of various human diseases. But the miRNA-neuroinflammation field is still naive (45).

In the present study, arsenic could neither decrease the CD200R1 mRNAs level similar to that reported with Rbfox and Kv1.1 and EGFR (46, 47, 48) nor increase the expression of CEBPβ. LPS, on the other hand, altered the expression of both CD200R1 and CEBPβ as expected (28, 29). These results indicated potential posttranscriptional regulation, and we focused on the possible role of miRNA, as they are prominent posttranscriptional regulatory molecules (31). The TaqMan low density miRNA array showed 15 significantly upregulated (≥1.5-fold) and 18 significantly downregulated (≤0.8-fold) miRNAs. The network analysis gave us a preliminary idea of the relationship between interacting molecules such as arsenic, TNF, IL-6, CD200R1, DNMT1, CD200, and the dysregulated miRNAs. But we did not find any direct or indirect relationship of CD200R1 with any miRNAs.

MiRNAs targets 30 to 80% of protein-coding genes (31) by binding to their 3′-UTR, coding sequence, or 5′-UTR (49). Our *in silico* analysis (50) predicted two potential binding sites of miR-129-5p in the 3′-UTR of mouse and human CD200R1 *via* incomplete pairing (51). Several miRNAs have been reported to induce microglial activation and neuroinflammation by altering the associated gene expression. miR-155 induces microglial activation by targeting SOCS1, and miR-146a represses the activation by targeting IRAK1/TRAF6. Similarly, miR-206 induces microglial inflammatory response, and miR-17 reduces the response by targeting NOX2/NOX4 (45, 49). MiR-129-5p has been studied for its role in various types of cancers (52, 53, 54), regulation of potassium channel (47), atherosclerosis and cardiovascular disease (54), regulation of neurogenesis (48), and synaptic scaling (46). Some reports have shown its anti-inflammatory role by targeting HMGB1, a potential TLR4 agonist in reperfusion injury and neuropathic pain in rodent models (55, 56). There is no information on the possible role of miR-129-5p in neuroinflammation. For the first time, we show that miR-129-5p plays role in regulating the expression of a surface receptor, which is intimately associated with neuroinflammation. Our study with miRNA mimics and inhibitors shows that expressions of miR-129-5p and CD200R1 are inversely related. There are two miRNAs, miR-297c-3p and miR-345-3p among arsenic-induced downregulated miRNAs. *In silico* analysis also predicted these two miRNAs to target CD200R1. The upregulated miRNAs are most likely to play direct role in translational repression by binding to 3′UTR of a particular gene; therefore we focused on miR-129-5p. However, interaction between CD200R1 and miR-297c-3p/miR-345-3p can be proven equally interesting.

The two predicted binding sites of miR-129-5p in the 3′-UTR of mouse CD200R1 mRNA were confirmed by generating a luciferase construct containing WT and mutated sequence in the miRNA-binding site. The significant reversal of the luciferase activity in the cells transfected with the mutant construct proves that both sites to be involved in miRNA binding (57, 58).

MiRNAs are reported to induce translational repression of almost one-third of mammalian mRNAs (59), by guiding them to the cytoplasmic processing bodies (P-bodies). P-bodies are the cytoplasmic foci formed of aggregation of many proteins, mRNA, and miRNA. It serves as the temporary mRNA storage depot or decay site, depending on the requirement (60, 61, 62, 63). Our study shows that arsenic and pre-miR-129 increased the p-body formation as detected by immunostaining one of its essential components, GW182 (64), and the p-bodies disappeared following GW182 siRNA treatment (65). We predicted that CD200R1 mRNA is included in the p-body after forming relatively weaker AU-rich base pairing with miR-129-5p (63). The increased p-body formation supports our prediction that miR-129-5p binds to 3′-UTR of CD200R1 mRNA and guides it to p-bodies. The p-body formation data was well matched with the mRNA degradation data. Pre-miR and arsenic were found to protect the CD200R1 mRNA following actinomycin D treatment (58), whereas GW182 siRNA allowed the mRNA degradation. Though statistically nonsignificant, there was a notable increase in the level of CD200R1 mRNA in pre-miR-129 and arsenic-treated group compared with control. It might be due to 72 h preincubation with pre-miR-129 and arsenic before Actinomycin D treatment, which resulted in the inclusion and storage of CD200R1 mRNA into P-bodies. The enrichment of miR-129-5p and CD200R1 mRNA in the GW182 immunoprecipitated samples (GW182 IP) confirmed that miR-129-5p guides the CD200R1 mRNA to p-bodies, thereby inducing translational repression of CD200R1. Therefore, the formation of p-body and the expression of CD200R1 are inversely correlated.

The next obvious query is how arsenic increases the level of miR-129-5p. The CpG islands of the miR-129-5p promoter are methylated in various cancers (52). Interestingly, hypermethylation of miR-129-5p CpG island was also observed in multidrug-resistant gastric cancer cell lines compared with parent cells (52). In contrast, bisulfite sequencing in the present study showed hypomethylation of CpG island of the miR-129-5p promoter. Arsenic-induced decrease in the expression of DNMT1 affects the *de novo* methylation and the maintenance of methylation status (37). Recently, it has been reported that CCCTC-binding factor, CTCF, is involved in the regulation of DNMT1 expression, and arsenic-mediated inhibition of CTCF is responsible for DNMT1 downregulation (40). Interestingly, CTCF is a DNA-binding protein with 11 zinc finger domain, out of which one is C3H1 type having three cysteine residues in cluster coordinated with a divalent zinc ion. Trivalent arsenic reduces the binding activity of CTCF by replacing zinc ion in C3H1-type zinc finger motifs (40, 66); therefore, it may results in the downregulation of DNMT1 expression as we see in the present study.

In addition to confirming the role of miR-129-5p in the posttranscriptional regulation of CD200R1 expression *in vitro*, we validated the same in the mouse model by injecting anti-miR-129 and CD200R1 siRNA in the brain (46). Anti-miR-129 reversed the arsenic-induced reduction of CD200R1 expression and cytokine levels, whereas anti-miR-129 could not reverse the effect of arsenic in siRNA-induced CD200R1 inhibited animals, which supports the proposed axis miR-129-5p→CD200R→TNFα/IL-6. The finding also instigates its potential therapeutic application in neuroinflammation (45, 51).

CD200R1 and miR-129-5p both are expressed in PBMCs (1, 3, 4, 53); therefore, the level of CD200R1 mRNA, as well as miR-129-5p, the level of CD200R1 and DNMT1 protein and mRNA of IL-6/TNF-α in the PBMCs of control and arsenic exposed human subjects have been checked. We observed results similar to animal experiments. Correlation analysis revealed that CD200R1 and DNMT1 are negatively correlated with miR-129-5p, and CD200R1 is positively correlated with DNMT1. The correlation analysis confirms that miR-129-5p also plays an essential role in the posttranscriptional regulation of CD200R1 expression in human PBMCs. Interestingly, the peripheral neuropathy observed in our study lies in line with the previous studies where the contribution of activated microglia (67) and arsenic (68) in neuropathy was defined.

**Figure 7:**
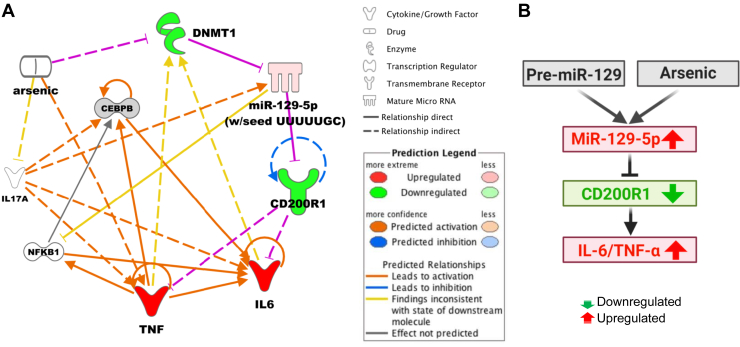
**Translational repression of CD200R1 in microglia.***A*, the plausible relationship between miR-129-5p and CD200R1 deduced from the results of the study is shown here in an IPA generated network with the new link shown in *pink*. The *symbols* are indicated in the legend. Significantly downregulated genes are in *green*, upregulated are in *red*. *Solid* and *dotted lines* indicate direct and indirect interactions, respectively. *B*, proposed pathway of posttranscriptional regulation of CD200R1 and its impact on neuroinflammation.

We propose a novel posttranscriptional regulatory mechanism for CD200R1 expression where miR-129-5p plays an indispensable role and controls neuroinflammation. Arsenic demethylates the CpG islands in the promoter of miR-129-5p by decreasing DNMT1, which in turn increases the level of miR-129-5p. miR-129-5p binds to the 3′-UTR of CD200R1, thereby induces translational repression, and the levels of the proinflammatory cytokine are elevated (Fig. 7). To conclude, antagonizing the miR-129-5p, a novel regulator of CD200R1 expression, represents a potential strategy for therapeutic intervention of neuroinflammation.

## Experimental procedures

### Reagents and antibodies

Sodium arsenite (SA) (NaAsO_2_), Percoll, cell culture medium (DMEM/F12), Papain, sodium dodecyl sulfate (SDS), acrylamide, bisacrylamide, bromophenol blue, Tween 20, EDTA, protease inhibitor cocktail, methanol (LC grade) were obtained from Sigma. Fetal bovine serum (FBS) was purchased from Cell Clone Inc. PVDF membrane, and chemiluminescence substrates were obtained from Merck-Millipore. The CD200R1 antibody (both mouse (AF2554) and human-specific) was purchased from R&D Systems. DNMT1 (sc-271729) and CD200 antibody (sc-71764) were procured from Santacruz biotechnology. N-2 supplement (17502048, Thermo), TaqMan Array Rodent MicroRNA A + B Cards Set v3.0, Megaplex RT Primers, Megaplex PreAmp Primers, TaqMan Universal PCR Master Mix (2X), mirVana miRNA Isolation Kit (AM1560), miR-129-5p & sno-202 TaqMan real-time assay kit, pre-miR-129 (assay ID: PM10195, ABI), anti-miR-129 (assay ID: AM10195, oligo sequence: 5′GCAAGCCCAGACCGCAAAAAG3′), *in vivo* ready CD200R1 siRNA, (cat: AM16830, ID: 181777, oligo sequences: Ass seq: AUAUGGUCGUAAUGAUUGGTT; Ss seq: CCAAUCAUUACGACCAUAUTT), *in vivo* negative control si RNA (cat: 4457287), HRP-anti-goat, HRP-anti-rabbit, Alexa Fluor 594 anti-rabbit (A-11037), Alexa Fluor 488 anti-goat (A-21210), DNA coimmunoprecipitation kit (14321D), and Procartaplex cytokine detection kit were obtained from Thermo Scientific. Beta-actin (ab8227, Abcam) and CD68 antibody (ab12512, Abcam) were procured from Abcam. GAPDH antibody (PG-27002) was obtained from puregene. DNA miniprep isolation kit, DNA bisulfite conversion kit, and Taq polymerase were purchased from Zymo Research. Tissue freezing medium was obtained from Leica. GW182 siRNA (sc45517) and pmyc-GFP-TNRC6A (plasmid for GW182, Cat. 41999, Addgene). A list of antibody and primers used in the study has been given in the supplementary information (Table S2).

### Animal husbandry and treatment

Six- to eight-week-old male BALB/c mice were procured from the CSIR–Indian Institute of Toxicology Research (CSIR-IITR) animal facility. All the protocols for this study were approved by the Institutional Animal Ethics Committee (IAEC) of CSIR-IITR, Lucknow, India, and all experiments have been carried out following the guidelines laid down by the committee for the purpose of control and supervision of experiments on animals (CPCSEA), Ministry of Environment and Forests (Government of India), New Delhi, India. Mice were housed at 25 °C with food and water supplied *ad libitum*. Animals were divided randomly into three groups (control, 0.038, and 0.38 mg/kg body weight NaAsO_2_ treatment group) by an investigator who is blind about treatment groups. Sodium arsenite solution was gavage-fed daily in arsenic treatment groups. Following 60 days treatment regimen, animals were sacrificed and used for various analyses. In another set, animals were divided into seven groups (Control, Sham control, Non-target/Scrambled control, Arsenic (0.38 mg/kg/bd wt), Arsenic + Anti-miR, Arsenic + Anti-miR + SiRNA, SiRNA). Sodium arsenite solution was gavage-fed daily in arsenic treatment groups for 60 days (60 d). Six days before sacrifice (54^th^ day), anti-miR-129 (0.25 nmol), CD200R1 siRNA (0.5 nmol) were intracerebrally injected alone and in combination. Sham control group received 2 μl nuclease free water. Total injection volume was 2 μl; each hemisphere received 1 μl. Detail of the stereotaxic method has been given later in this section.

### Human sample collection

The work with human samples was performed following the Declaration of Helsinki. Protocols for this study were approved by the Institutional Human Ethics Committee (IHEC) of CSIR-IITR in compliance with the Certificate of Approval of Institutional Human Ethics Committee (CAIHEC). For arsenic-exposed human sampling, Murshidabad district in the state of West Bengal, India, has been selected, where a high level of arsenic contamination is prevalent. For control human sampling, Lucknow district in the state of Uttar Pradesh, India, has been chosen, where the arsenic concentration reported below the WHO permissible limit. Blood was drawn from arm vein of control and symptomatic individuals showing clear raindrop pigmentation, collected in heparinized vacutainer tubes and kept on ice. Details of the parameters enquired during sampling are given in a table (Table S1).

### Isolation of primary microglia and treatment

Neonatal microglia were isolated from postnatal day 0 to 3 old pups following the protocol published from our lab earlier (35). Briefly, cortices were dissected from the brain and homogenized using a 5 ml syringe to form mixed glial cell suspension. Cells (0.4 × 10^6^) were seeded in 12-well plates in DMEM/F12 medium supplemented with 10% FBS and 1% pen-strep followed by media replacement after 48 h. The culture medium was replaced every fourth day until full confluency at around 30 days. Mix glial culture was incubated for 45 min in serum-free media and trypsin solution (1:1 ratio), followed by the removal of floating cells. The attached cells are the pure microglia that were used for further experiments. Primary adult microglia were isolated following the protocol published earlier from our lab (36) Briefly, brain samples were chopped and enzymatically digested with papain (20 U/ml) at 37 °C for 20 min. Resulting digested tissue was mixed with 30% isotonic percoll and centrifuged at 500*g* for 20 min at 20 °C. Isolated cells were immediately processed for RNA isolation using the mirVana kit or cultured at a density of 5 × 10^4^ cells/well in 96-well culture plate for cytokine measurement in the resulting culture supernatant. For *in vitro* arsenic treatment to primary microglia, 500 nM concentration was used in all the experiments.

### Cell line maintenance

Mouse microglia cell line (BV2) and human microglia cell line (CHME3) were cultured in DMEM/F12 medium supplemented with 10% FBS and 1% pen-strep at 37 °C with 5% CO_2_. BV2 cells were used in RNA immunoprecipitation studies, whereas CHME3 cells were used to check CD200R1 expression by Western blot analysis and immunocytochemistry.

### Preparation of cell/tissue lysate and Western blot analysis

Cells were washed in cold PBS and scrapped in 30 μl of cell lysis buffer (20 mM Tris–HCl pH-8, 137 mM NaCl, 10% glycerol, 1% Triton X-100, 2 mM EDTA, and protease inhibitor cocktail). Cells from three wells were pooled together in a 1.5 ml tube and kept on shaking at 4 °C for 30 min. For tissue lysate preparation, the brain was dissected and homogenization in cell lysis buffer. Finally, the cell/tissue lysate was centrifuged at 14,000*g* for 15 min at 4 °C, and the supernatant collected and used for Western blot analysis. Protein concentration was estimated by BCA Kit (23235, Thermo). Twenty micrograms of protein of each sample was run on 10% SDS-PAGE, transferred to PVDF membrane, and probed with the desired primary antibody diluted in TBS with tween-20 (0.05%) followed by incubation with fluorescent-tagged secondary antibody. Blots were observed in a gel documentation system (G-box H-16, Syngene and Amersham Imager 600, GE Healthcare) using SuperSignal West Femto Maximum Sensitivity substrate (34094), and densitometric analysis was performed by Image-J software.

### Taqman low-density array (TLDA) for miRNA

The expression of 641 miRNAs was studied by Taqman Low-Density Arrays Pool A and pool B (TaqMan Rodent MicroRNA Set Cards v3.0: Part no. 4398979 for pool A contains 317 and 4455449 for pool B contains 324 miRNA primers) following the manufacturer’s instruction. In brief, total RNA was isolated from *in vitro* arsenic-treated (72 h) primary neonatal microglia using mirVana kit. RNA (300 ng) was subjected to reverse transcription (RT) by using megaplex RT primers of pool A and pool B. After reverse transcription, preamplification was carried out using TaqMan preamp master mix and megaplex preamp primers of pool A (part no. 4399203) and pool B (part no. 4444308). Finally, for a single plate, 450 μl of Taqman universal PCR master mix was mixed with 9 μl of preamp product along with 441 μl nuclease-free water. In each port of TLDA plates, 100 μl from the abovementioned master mixture was added, and after sealing and spinning, the plates were loaded on a Quant studio 12K Flex Real-Time PCR system (Thermo). Relative quantification was done using ^−ΔΔ^Ct method considering the levels of endogenous controls with expression suite online software (Thermo).

### Real-time PCR of miRNAs

The total mRNA was isolated using the mirVana kit from microglia. The level of miR-129-5p was detected using specific TaqMan microRNA assays (part number 4373068, Applied Biosystems) and TaqMan Universal PCR Master Mix, No AmpErase UNG (part number 4324018; Applied Biosystems) following the manufacturer’s instruction in aquantstudio 6 flex real-time PCR system. The level of miRNA expression was measured by relative quantification performed using ^∼ΔΔ^Ct method where Ct values of miR-129-5p were normalized to sno202 RNA Ct values from the same sample for each group.

### Real-time PCR

Total RNA was isolated either from brain samples (100–200 mg) or cell pellets using Trizol reagent (Invitrogen). The concentration was determined using a Nanodrop spectrophotometer (Thermo). cDNA was synthesized using a high-capacity cDNA reverse transcription kit (part number 4331182) (ABI). The qRT-PCR was run in a quantstudio 6 flex real-time PCR system (Thermo) using SYBR green master mix (PGK022A, Puregene) and specific forward-reverse primers. Mouse β-actin was used for relative quantification following ^∼ΔΔ^Ct method.

### Immunocytochemistry and immunohistochemistry

Microglia were grown on glass coverslips and treated as per requirement. Cells were washed with PBS and fixed with 4% paraformaldehyde followed by incubation with blocking buffer (1 PBS + 2% FBS + 0.05% Tween-20) for 1 h at room temperature. The desired primary antibody added to the cells and incubated overnight at 4 °C. Cells were washed and further incubated with fluorescence tagged secondary antibody for 2 h at room temperature. Finally, coverslips were mounted on a glass slide with DAPI containing antifade mounting medium (Vector lab) and observed under a fluorescence microscope. For immunohistochemistry, 12 μm coronal sections of brain tissue were cut from paraformaldehyde perfused brain using cryotome and kept on silane-coated slides. A similar staining protocol followed as described for immunocytochemistry and observed under Nikon Eclips fluorescence microscope (Nikon Instrument Inc). For quantifying CD200R1 staining, fluorescent Images was converted to 8 bit grayscale image followed by thresholding using National Institutes of Health ImageJ software (NIH, https://imagej.nih.gov/ij/). Finally, percentage area occupied by CD200R1 and DAPI staining is quantified for each image using analyzed particle tool in ImageJ, and data are represented as percentage CD200R1 staining of DAPI area (69).

### P-body immunostaining

Neonatal primary microglia were cultured on coverslip in 12-well plate followed by treatment with arsenic (500 nM), pre-miR129 (10 nM), GW182 siRNA (30 nM), GW182 + Arsenic, and GW182 siRNA (30 nM) + pre-miR-129 for 72 h. After treatment, cells were immunostained with the GW182 antibody, as described in the Immunocytochemistry section. Images were captured in confocal microscope (Zeiss) and number of p-body (GW182-associated fluorescent dots) counted. Primary *ex vivo* microglia were also stained with GW182 antibody to check formation of p-body following *in vivo* arsenic exposure. Microglia (*ex vivo* microglia) were isolated from control and arsenic-exposed mouse, seeded in 8-well chambered slides (1 × 10^5^ cells in each well) and allowed to adhere for 1 h. Cells were fixed in chilled methanol and immunostained for P-bodies using GW182 antibodies. As the *ex vivo* microglia lose its normal flattened morphology and become round following isolation, therefore counting number of p-body is not possible. Therefore, we have scored the GW182 fluorescence in each cell and expressed Fl/unit area/cell.

### Transfection of pre-miR-129, anti-miR-129, and GW182

Neonatal primary microglial (60,000 cells/well in a 12 well culture plate) were transfected with pre-miR-129 (10 nM) and anti-miR-129 (100 nM) using siPORT NeoFX transfection agent. Briefly, transfection reagent was mixed with opti-MEM, followed by the addition of pre-miR-129 or anti-miR-129 kept at room temperature for 10 min. Hundred microliters of resulting transfection complex was added to microglia for 72 h and processed for Western blot analysis or qRT-PCR. For the transfection of GW182, BV2 cells were transfected using FuGENE Transfection Reagent (E2691, Promega). Fugene was mixed with Opti MEM, and the mixture was incubated for 5 min. Then, plasmid (3 μg) was added into the mixture, incubated for 15 min, and the transfection complex was added into the cells in a T75 flask. Transfection efficiency was checked by Western blot analysis.

### Bioinformatic analysis

Significant fold changes of miRNAs calculated from ExpressionSuite software (Thermo) were visualized as a heat map, and the cluster was generated with Cluster 3.0 software (http://bonsai.hgc.jp/∼mdehoon/software/cluster/software.htm). Fold changes of the miRNAs were then submitted to Ingenuity Pathway Analysis (IPA, QIAGEN Inc https://www.qiagenbioinformatics.com/products/ingenuity-pathway-analysis) for core analysis based on experimentally observed and predicted data resourced from the Ingenuity Knowledge Base. The top canonical pathways, diseases and functions, and gene networks that are most significant to microarray studies were identified, and differentially expressed genes in specific diseases and functions were categorized. These genes were then used to generate functional networks between miRNAs and their target molecules. For miRNA target prediction, initially, TargetScan (http://www.targetscan.org/mmu72/) was used, followed by RNA22 (https://cm.jefferson.edu/rna22/) and RNA hybrid (https://bibiserv.cebitec.uni-bielefeld.de/rnahybrid/) software for obtaining free energy and structure. For CpG site identification and primer designing, Methprimer software (http://www.urogene.org/cgi-bin/methprimer/methprimer.cgi) was used by providing 1000 bp fragment of miR-129-2 gene upstream of the transcription start site.

### 3′-UTR luciferase reporter assay

WT and mutant (Mut1 and Mut2) plasmid of 3′UTR of CD200R1 mRNA were customs designed from Thermo (Thermo Fisher Scientific). WT and mutant 3′UTR were subcloned into the MluI and SpeI site of the pMIR-report vector. These products are referred to as pMIRCD200R1-WT for WT and pMIR-CD200R1-Mut 1 & pMIR-CD200R1-Mut 2 for the WT and mutant 3′UTR from the CD200R1 gene, respectively. HEK cells (5000 cells/well) were seeded at in 96-well tissue culture plate along with siPORT NeoFX transfection agent containing pre-miR-129 for 8 h. After transfection, the medium was removed, and cells were rinsed followed by transfection with pMIR-reporter, pMIR-CD200R1-WT, or pMIR-CD200R1-Mut1/2 and pMIR-galactosidase vectors using Fugene transfection reagent. After 48 h transfection, cells were harvested and lysed in 200 μl of lysis buffer, and the lysates were assayed for luciferase and galactosidase activity following standard protocols.

### mRNA stability assay

In order to check CD200R1 mRNA stability, neonatal primary microglial (60,000 cells/well of 12 well culture plates) were treated with arsenic (500 nM), pre-miR-129 (10 nM), GW182 siRNA (30 nM), GW182 siRNA + Arsenic, and GW182 siRNA + pre-miR-129 for 72 h followed by addition of actinomycin D (ActD; 10 μg/ml) to halt transcription. At 2 h post-ActD addition, cells were harvested and RNA isolated. Subsequently, CD200R1 mRNA levels were determined using qRT-PCR described earlier in the Experimental procedures section.

### RNA immunoprecipitation

For RNA immunoprecipitation, GW182 was overexpressed in BV2 cells by transfecting pmyc-GFP-TNRC6A plasmid (2.5 μg) for 24 h followed by treatment of arsenic (1 μM) and pre-miR-129 for another 72 h and 48 h, respectively. Although we have used 500 nM arsenic for *in vitro* treatment to primary cells, but could not find any upregulation of miR-129-5p in BV2 cells with the same dose; therefore, we have used 1 μM arsenic to treat BV2 cells. BV2 cells (1 × 10^6^) were seeded in T75 flasks and treated as required. After completion of the treatment, cells were fixed with 0.75% paraformaldehyde for 5 min, followed by neutralization in 125 mM glycine for an additional 5 min. Cells were scraped and lysed in ice. We have started the immunoprecipitation with 800 μg cell lysate and followed the protocol supplied with the Dynabeads CO-IP kit (Thermo). Finally, the immunoprecipitated pellet was resuspended in nuclease-free water and used to measure the level of CD200R1 mRNA and miR-129-5p RNA qRT-PCR.

### Bisulfite sequencing

Genomic DNA (gDNA) was extracted from neonatal primary microglia following 72 h arsenic treatment using Quick-DNA Miniprep Plus Kit (D4068, Zymo Research). One microgram gDNA was bisulfite converted using EZ DNA Methylation-Gold Kit (D5005, Zymo Research) following the manufacturer protocol. After bisulfite conversion, a 503 bp long fragment containing 15 CpG sites was amplified using forward primer 5′-AAAAAGAAATGTGAGTTTTTTTT-3′ and reverse primer 5′-AAAACTAAATCTCCCCGACG-3′ and sequenced. We outsourced the sequence service to Integrated DNA Technologies Inc (IDT). For the quantification of CpG methylation, CpG viewer software (http://dna.leeds.ac.uk/cpgviewer/) was used.

### Intracerebroventricular injection of CD200R1 siRNA and anti-miR-129

In order to confirm the role of miR-129-5p in regulating the expression of CD200R1 and inflammation, we inhibited CD200R1 and miR-129-5p by intracerebral injection of CD200R1 siRNA and anti-miR-129 using the stereotaxic technique. Briefly, intraperitoneal injection of ketamine and xylazine (60 and 20 mg/kg body weight, respectively) was given to deeply anesthetize each mouse before shaving its head and positioning it in the stereotaxic frames (Stoelting Co). On the dorsal side of the head, a midline scalp incision was performed in order to expose the skull and visualize bregma and lambda. *In vivo* ready siRNA for CD200R1 (0.5 nmol in 2 μl/brain, 1 μl in each hemisphere, cat: AM16830, thermo) and anti-miR-129 (0.25 nmol in 2 μl/brain, 1 μl in each hemisphere) was injected in each cortical hemisphere at the rate of (0.5 μl/min) into the cerebral cortex using a 10-μl Hamilton syringe at the following coordinates: 0.6 mm posterior, 1.5 mm lateral, and 1.3 mm dorsal with respect to bregma, once at 54th day of arsenic exposure, and dissected 6 days after injection (70). In the anti-miR and siRNA coexposed group, the concentration was adjusted such that the injection volume remains 2 μl/brain. The scrambled control group received negative control siRNA (0.25 nmol 2 μl/brain, *in vivo* negative control siRNA, cat: 4457287, thermo) in the same manner. The sham control group was treated with sterile nuclease-free water that followed the same procedure. The whole brain samples were used to detect CD200R1 expression by Western blot and immunohistochemistry. At the same time, microglia were also isolated and cultured for 18 h *ex vivo*. Levels of IL-6 and TNF-α were measured in the culture supernatant using a multiplex cytokine detection kit (Procartaplex, Thermo).

### Detection of cytokine

Microglia isolated from adult mice (*ex vivo* microglia) were seeded at a density of 5 × 10^4^ cells in 100 μl DMEM/F12 medium. After culturing the cells for 18 h, culture supernatant was collected for measuring IL-6 and TNF-α using the procartaplex mouse cytokine assay kit (Thermo) following the manufacturer’s protocol. The infrared (IR) fluorescence associated with different antibody-coated magnetic beads was read in a multiplex reader (Bioplex MAGPIX multiplex reader, BioRad). The level of cytokine was calculated from a standard curve of that particular cytokine and expressed as pg/ml.

### Statistical analysis

Analysis of two groups was performed by unpaired Student’s *t* test, whereas, for more than two groups, one-way analysis of variance (ANOVA) was performed, followed by Newman–Keuls post-hoc test by GraphPad Prism. Correlation analysis was also performed in GraphPad Prism. A value of *p* < 0.05 was considered statistically significant.

## Data availability

All data generated for this study are contained within the manuscript. For further queries, corresponding author D. G. may be contacted.

## Supporting information

This article contains supporting information.

## Conflict of interest

The authors have declared that no conflict of interest with the contents of this article.
