# Supplementary material for: MicroRNA-129-5p-regulated microglial expression of the surface receptor CD200R1 controls neuroinflammation
Source: J Biol Chem. 2021 Dec 22;298(1):101521. doi: 10.1016/j.jbc.2021.101521 (PMC8762073; doi:10.1016/j.jbc.2021.101521)
Supplement: Supplemental Figures S1–S6 and Tables S1 and S2 [file mmc1.pdf]

MicroRNA-129-5p-regulated microglial expression of the surface receptor CD200R1 controls neuroinflammation

Running title**:** miR-129-5p and CD200R1 in neuroinflammation

Vikas Singh[1,2,6], Shaivya Kushwaha[1,2,¶], Jamal Ahmad Ansari[1,2,¶], Siddhartha Gangopadhyay[2,3], Shubhendra K. Mishra[1], Rajib K. Dey[1,2], Ashok K. Giri[4], Satyakam Patnaik[2,5], Debabrata Ghosh[1,2*].

[1] Immunotoxicology Laboratory, Food, Drug & Chemical Toxicology Group and Nanomaterial Toxicology Group, CSIR-Indian Institute of Toxicology Research (CSIR-IITR), Vishvigyan Bhawan, 31, Mahatma Gandhi Marg, Lucknow, Uttar Pradesh 226001, India.

[2] Academy of Scientific and Innovative Research (AcSIR), Ghaziabad 201002, India.

[3] Developmental Toxicology Laboratory, Systems Toxicology & Health Risk Assessment Group, CSIR-Indian Institute of Toxicology Research (CSIR-IITR), Vishvigyan Bhawan, 31, Mahatma Gandhi Marg, Lucknow, Uttar Pradesh 226001, India.

[4] Molecular Genetics Division, CSIR-Indian Institute of Chemical Biology, 4, Raja Subodh Chandra Mallick Rd, Poddar Nagar, Jadavpur, Kolkata, West Bengal 700032, India.

[5] Water Analysis Laboratory, Nanomaterial Toxicology Group, CSIR-Indian Institute of Toxicology Research, Lucknow, Uttar Pradesh 226001, India

[6] present address: Department of Neurosciences, Lerner Research Institute, Cleveland Clinic, Ohio 44195, USA

*Debabrata Ghosh
Email: Debabrata.Ghosh@iitr.res.in; debabrataghosh78@gmail.com
ORCID: 0000-0002-6571-304X

[¶] these authors contributed equally

Keywords
Microglia, miR-129-5p, CD200R1, arsenic, miRNA, neuroinflammation, Cytokine, neuroimmunology

Conflict of interest
The authors have declared that no conflict of interest exists.

Supporting information:

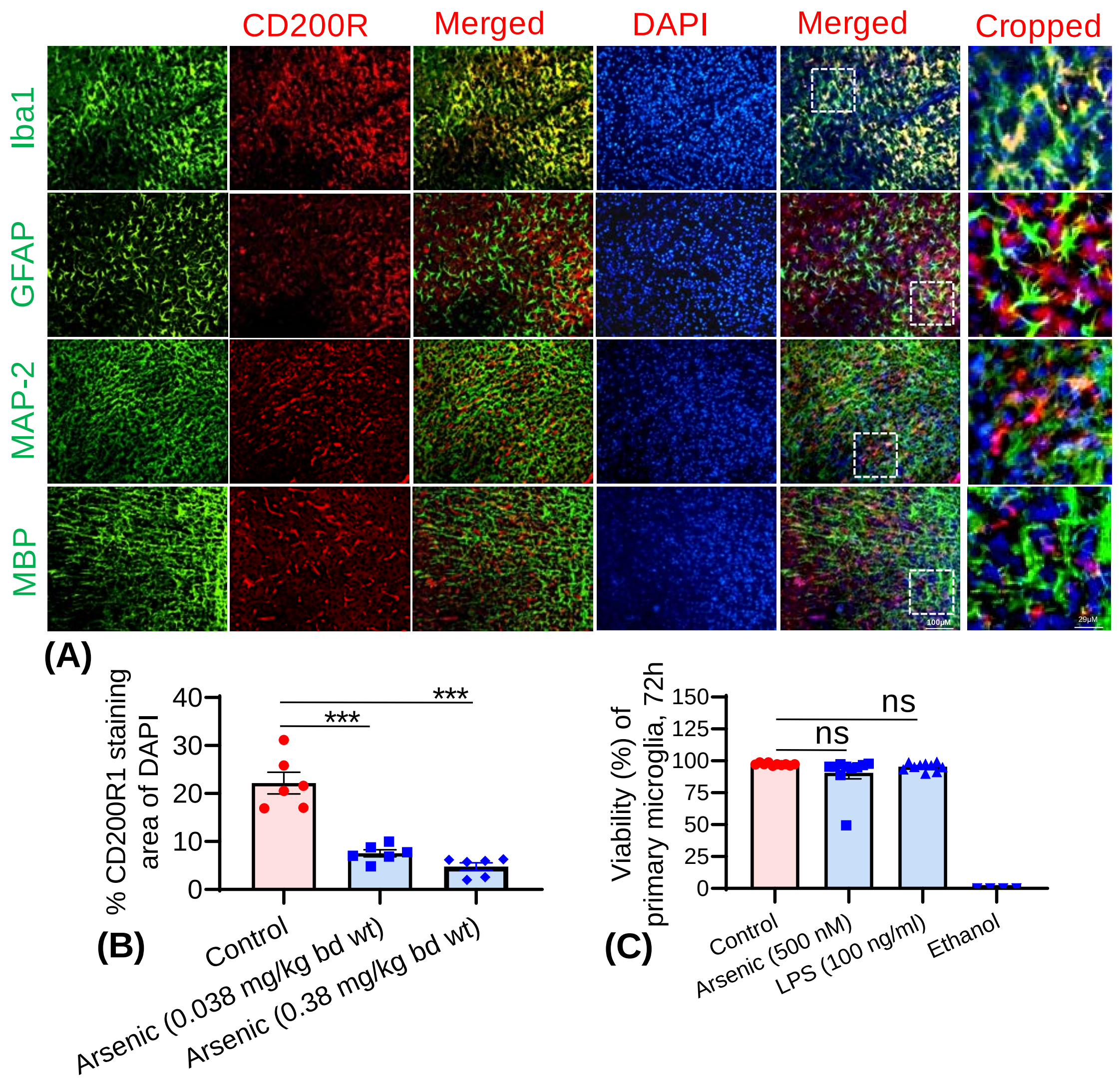

Figure S1. Immunostaining of mouse brain sections, CD200R1 associated fluorescence and viability of primary microglia. (A) Mouse brains were isolated and processed for cryosectioning. Brain cryosections were co-immunostained in the following combinations; CD200R1 and Iba1 (micoglia marker), CD200R1 and GFAP (astrocyte marker), CD200R1 and MAP-2 (Neuronal marker) and CD200R1 and MBP (oligodendrocyte marker). CD200R1 was co-localized only with Iba1, whereas co-localization of CD200R1 with any other marker was not evident. Scale bar=100μm for uncropped images and 29μm for the cropped images. **(B)** Levels of CD200R1 associated fluorescence in mouse brain sections following arsenic exposure. Animals were exposed to arsenic (0.038 and 0.38 mg/kg bd. wt.) for two months and sacrificed. Brains were processed for cryosectioning and immunofluorescence staining with CD200R1 antibody (n=3 mice/group). Randomly two fields were selected from each animals and CD200R1 associated fluorescence was quantitated in "Image J" and expressed as histogram. **(C)** Viability of primary neonatal microglia following arsenic and LPS exposure. Primary neonatal microglia were seeded in 12 well culture plates and treated with arsenic (500 nM) and LPS (100 ng/ml) for 72h. Following incubation, viability was assessed by trypan blue dye exclusion test. Viability was expressed as mean±SD (n=2, experiment was repeated twice, two fields for ethanol group and five fields were considered for other groups in each experiment). There was no significant alteration observed in any of the groups. 'n' denotes the number of independent experiments. Bar graphs represent mean±SEM. '*p*' denotes the level of significance in comparison to control; *$p<0.05$, **$p<0.01$, ***$p<0.001$; ns: non-significant.

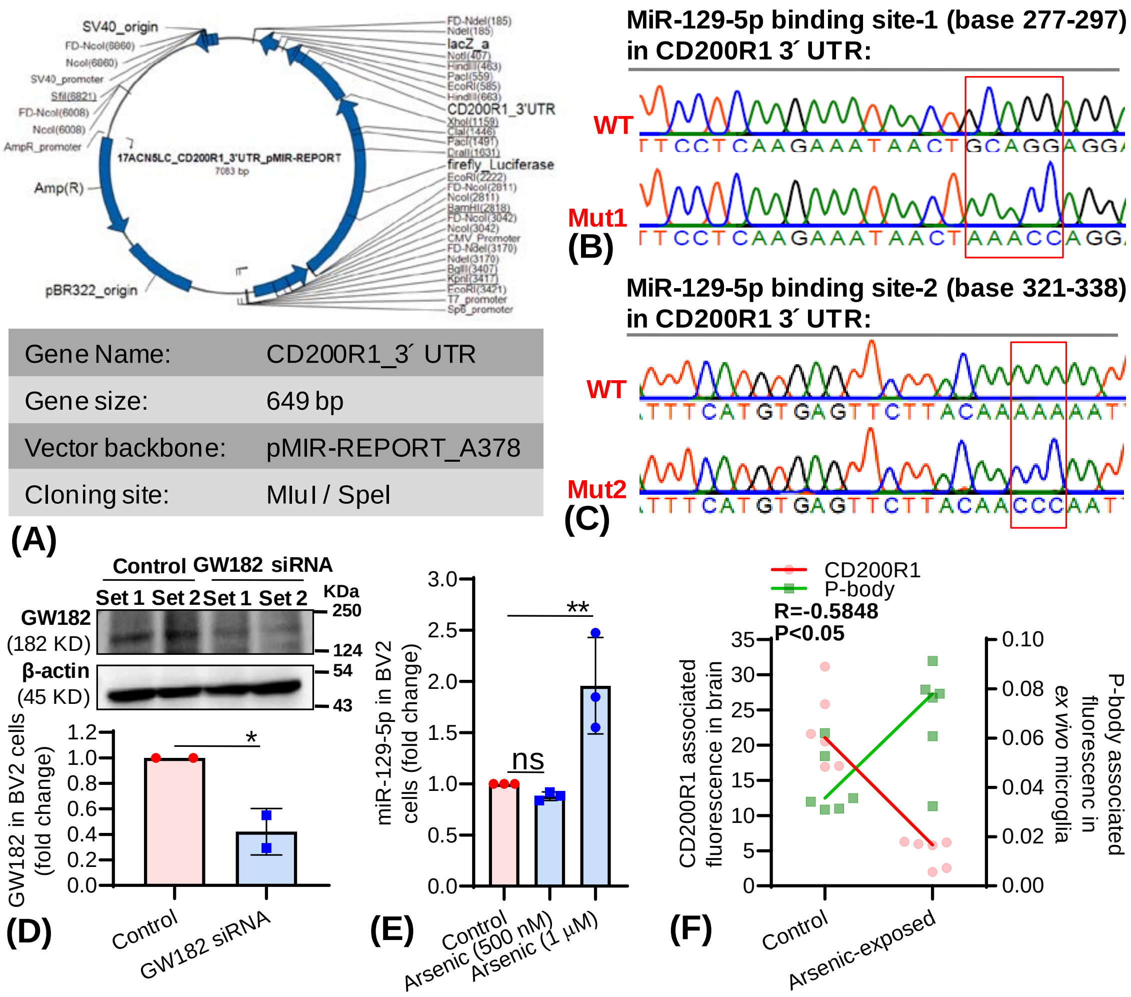

Figure S2. CD200R1-3´UTR-pMIR-REPORT plasmid constructs, inhibition of GW182 by siRNA, miR-129-5p in BV2 cells and correlation of P-body and CD200R1 expression. (A) Detailed map of the CD200R1-3´UTR-luciferase plasmid. **(B)** Chromatogram of wild type (WT) and mutant1 (Mut1) sequence and, **(C)** chromatogram of wild type (WT) and mutant2 (Mut2) sequence. Mutant sequences are shown in the box with corresponding WT sequences. **(D)** Inhibition of GW182 by siRNA. BV2 cells were transfected with GW182-siRNA for 72h. Cells were harvested and analyzed for the expression of GW182 in the cell lysate by western blot (n=2). Almost 60% inhibition of basal GW182 expression was observed following siRNA transfection. **(E)** BV2 cells were (0.15×10^6 cells) exposed to arsenic (500 nM and 1 µM) for 72h (n=2). Following treatment cells were harvested, RNA isolated, and checked for the level of miR-129-5p. A significant increase in miR-129-5p level was observed in exposure to 1 µM arsenic, whereas, 500 nM arsenic did not induce any change. ‘n’ denotes the number of independent study for *in vitro* study. **(F)** Fluorescence associated with CD200R1 in brain sections and P-body associated fluorescence in *ex vivo* microglia was measured using “Image J”. Using the CD200R1 and P-body fluorescence Pearson correlation analysis was performed in GraphPad Prism. The analysis revealed an inverse relation between the two parameters. ‘R’ denotes Pearson correlation coefficient. Bar graphs represent mean±SEM. ‘*p*’ denotes the level of significance in comparison to control; *$p<0.05$, **$p<0.01$, ***$p<0.001$; ns: non-significant.

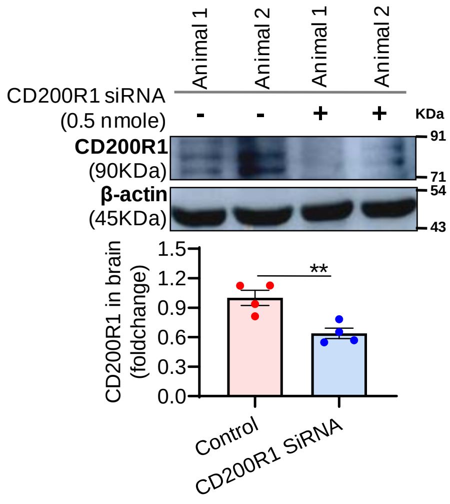

Figure S3. SiRNA-induced inhibition of CD200R1 in mouse brain. To generate a loss-of-function model CD200R1 siRNA was stereotaxically injected into brain of mouse (n=4 mice/ group). On the 6th day following injection mice were sacrificed and expression of CD200R1 was checked by western blot analysis. The expression of CD200R1 was observed to be ~0.63 fold in CD200R1 siRNA -treated group compared to control. Bar graphs represent mean±SEM. '*p*' denotes the level of significance in comparison to control; **p*<0.05, ***p*<0.01, ****p*< 0.001; ns: non-significant.

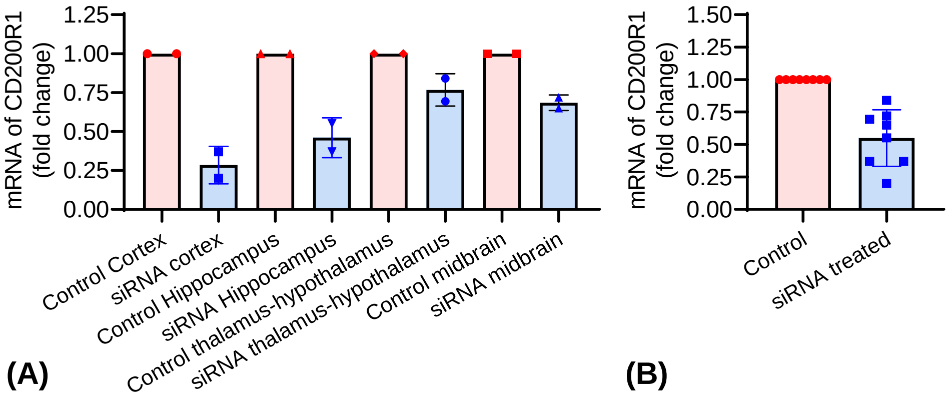

Figure S4. Anatomical region wise inhibition of CD200R1 mRNA following intracerebroventricular injection of CD200R1 siRNA. Control mice were stereotaxically injected as described in experimental procedures section. On the 6th day following injection mice were sacrificed, different anatomical regions were separated (Cortex, Hippocampus, Thalamus-hypothalamus and Midbrain) and expression of CD200R1 was checked by qRT-PCR and expressed as fold change over control. **(A)** Level of CD200R1 mRNA in different anatomical location of brain. (**B**) Mean value of CD200R1 mRNA fold change calculated together taking all the individual value of different region. n=2 animals in each of control and siRNA-treated group.

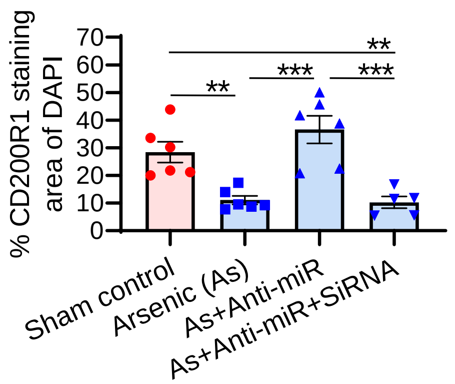

Figure S5. CD200R1 associated fluorescence in mouse brain section. Animals were exposed to arsenic (0.38 mg/kg bd. wt.) for two months, and in the last week of exposure, animals were treated with CD200R1 siRNA and anti-miR-129 intracerebrally by stereotaxic method and sacrificed on 60th day. Brains were processed for cryosectioning and immunofluorescence staining with CD200R1 antibody. (n=3 mice in group). Randomly two fields were selected from each animals and CD200R1 associated fluorescence was quantitated in "Image J" and expressed as histogram. Bar graphs represent mean±SEM. '*p*' denotes the level of significance in comparison to control; **p*<0.05, ***p*<0.01, ****p*< 0.001; ns: non-significant.

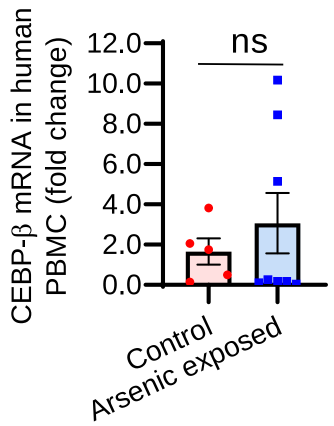

Figure S6: Level of CEBP-β mRNA in human PBMCs. PBMCs were isolated from the blood of unexposed (n=5 individuals) and arsenic-exposed symptomatic individuals (n=8 individuals). RNA was isolated, and the level of CEBP-β mRNA was measured by real-time PCR. A non-significant (ns) (*p*=0.8329) increase in the mRNA of CEBP-β was observed in arsenic-exposed group. Bar graphs represent mean±SEM.

Table S1. Detail of human sampling parameters. Table shows the detailed parameters of human sampling and the clinical features observed. M: Male; F: Female; RO: RO water; TWW: Tube Well Water; TW: Tap Water, NA: Not Applicable; L: Liter; Y: Years. ***** Mild neuropathy symptoms: Numbness, Muscle cramp, Pain. # High neuropathy symptoms: Numbness, Muscle cramp, Pain, paresthesia, Vibration joint sense, muscle wasting.

	Control population					Arsenic-exposed population								
Sample	C1	C2	C3	C4	C5	A1	A2	A3	A4	A5	A6	A7	A8	A9
Gender	M	F	M	M	M	M	M	F	F	Fe	M	M	M	M
Age	55	31	52	47	45	60	57	55	NA	48	N/A	78	48	70
Source of drinking water	RO	RO	RO	TWW	TWW	TW	TW	TWW /TW	TW	TWW /TW	TW	TW	TW	TWW /TW
Daily intake (L)	5	5	4	5	4	4	4	3	3	3	3	2	4	3
Duration of contaminated water drinking (Y)	NA	NA	NA	NA	NA	16	14	14	14	14	12	16	14	30
Raindrop Pigmentation	NA	NA	NA	NA	NA	Yes	Yes	Yes	Yes	Yes	Yes	Yes	Yes	Yes
Keratosis	NA	NA	NA	NA	NA	Yes	Yes	No	No	No	No	No	Yes	Yes
Peripheral neuropathy	No	No	No	No	No	No	Mild*****	No	High#	Mild	No	Mild	Mild	Mild

Table S2: List of antibodies and primers.

LIST OF ANTIBODIES				
**Antibodies**	**Reactivity**	**Vendor**	**Catalogue No.**	**Dilution**
CD200R1	Mouse	R&D Systems	AF2554 (Lot: UVI0112081 and UVI0118081)	WB:1:1000<br>ICC: 1:100<br>IHC:1:100
CD200R1	Human	R&D Systems	AF3414	WB: 1:3000<br>ICC:1:200
DNMT1	Human/ Mouse	Santa Cruz Biotechnology	sc-271729	WB:1:3000
Beta Actin	Human/ Mouse	Abcam	ab8227	WB:1:5000
GAPDH	Human/ Mouse	Puregene	PG-27002	WB:1:5000
GW182 (4B6)	Mouse	Santa Cruz Biotechnology	sc-56314	ICC:1:100<br>IP: 5 µg per sample
GW182 (A-8)	Mouse	Santa Cruz Biotechnology	sc-377006	WB: 1:5000
Alexa Fluor 594 anti-rabbit secondary	-	Thermo Scientific	A-11037	IHC:1:500
Alexa Fluor 488 anti-goat secondary	-	Thermo Scientific	A-21210	ICC:1:500
Alexa Fluor 488 anti-mouse secondary	-	Thermo Scientific	A-11029	ICC:1:500

LIST of qRTPCR PRIMERS			
**Species**	**Target Gene**	**Forward primer 5'>3'**	**Reverse primer 5'>3'**
Mouse	CD200R1	AGGAGGATGAAATGCAGCCTTA	TGCCTCCACCTTAGTCACAGTATC
	C/EBP-β	AAGCTGAGCGACGAGTACAAGA	GTCAGCTCCAGCACCTTGTG
	GAPDH	AGTGGCAAAGTAGAGATT	GTGGAGTCATACTGGAACA
	β-actin	CAACGAGCGGTTCCGATG	GCCACAGGATTCCATACCCA
Human	CD200R1	GACCAGAGAGGGTCTCACCA	TTGAAGCGGCCACTAAGAAG
	C/EBP-β	GACAAGCACAGCGACGAGTA	AGCTGCTCCACCTTCTTCTG
	GAPDH	CCACATCGCTCAGACACCAT	TGACCAGGCGCCCAATA
	18s rRNA	TGCCATGTCTAAGTACGCACG	TTGATAGGGCAGACGTTCGA
	β-actin	GTCATTCCAAATATGAGATGCGT	GCTATCACCTCCCCTGTGTG
	IL-6	TGCAATAACCACCCCTGACC	GTGCCCATGCTACATTTGCC
	TNF-α	GCCACCACGCTCTTCTGT	GGCTACGGGCTTGTCACTC
